# The effect of nutrition and reproductive health education of pregnant women in Indonesia using quasi experimental study

**DOI:** 10.1186/s12884-021-03676-x

**Published:** 2021-03-04

**Authors:** Tria Astika Endah Permatasari, Fauza Rizqiya, Walliyana Kusumaningati, Inne Indraaryani Suryaalamsah, Zahrofa Hermiwahyoeni

**Affiliations:** 1 Department of Nutrition, Faculty of Medicine and Health, Universitas Muhammadiyah Jakarta, Central Jakarta, 10510 Indonesia; 2 Central of the National Population and Family Planning Agency, East Jakarta, 13650 Indonesia

**Keywords:** Maternal, Nutrition education, Pregnant women, Reproductive health, Stunting

## Abstract

**Background:**

Almost one-third of children under 5 years old in Indonesia suffer from stunting. Stunting can be prevented optimally during pregnancy as the initial phase of the first 1000 days of life. This study aims to determine the effect of nutrition and reproductive health education of pregnant women in Bogor Regency, Indonesia.

**Methods:**

A quasi-experimental study was conducted among 194 pregnant women from August to November 2019. The pregnant women were randomly selected from four different villages in Bogor Regency. The intervention group (*n* = 97) received 2 h of nutrition and reproductive health education in small groups (four or five mothers per group) every 2 weeks for 3 consecutive months. This interactive education was given by facilitators using techniques such as lectures, role-playing, simulation, and games. The control group (*n* = 97) received regular health care services. A structured questionnaire was applied to collect data consisting of maternal characteristics, nutritional and reproductive health knowledge, attitudes, and practices in the intervention and control groups. Data were analysed using t-test and chi-square analysis.

**Results:**

Pregnant women in the intervention group indicated a significant increase in knowledge, attitudes, and practices regarding nutrition and reproductive health after receiving education. The pre-test and post-test mean scores in the intervention group were 55.1 and 83.1 for overall knowledge, 40.2 and 49.0 for attitudes, and 36.2 and 40.2 for practices, respectively. In the control group, there was no significant difference between the pre-test and post-test mean scores for these three variables. There was a significant difference (*P* < 0.001) in the post-test mean between the intervention group and the control group, but the difference was not significant (*P* > 0.05) in the pre-test.

**Conclusion:**

Providing nutrition and reproductive health education through small groups with interactive methods improves the knowledge, attitudes, and practices of pregnant women. This intervention has the potential to be replicated and developed for large-scale implementation by optimising collaboration between government, non-governmental organizations, and maternal and child health service providers.

## Introduction

Reducing stunting is part of the World Health Organization (WHO) Sustainable Development Goals (SDGs) [1]. In many countries, interventions to reduce stunting have been implemented during pregnancy [2, 3]. Poor maternal reproductive health and nutrition during pregnancy has lifelong impacts on the health of the offspring [4]. Furthermore, inadequate infant and child feeding practices, repeated infection, and inadequate psychosocial stimulation in the first 1000 days of a child’s life strongly contribute to stunted growth and development [5, 6]. Stunting reflects shortness for age, is a well-established risk marker of growth failure, and is measured by a height-for-age z-score of more than two standard deviations below the WHO Child Growth Standards median [7]. This chronic malnutrition is related to many indices of functional impairment, including cognitive and physical development, metabolic disorders that carry an increased risk of degenerative diseases, and socio-emotional development [8–11]. These serious health problems contribute to high health care costs of a country; therefore, effective prevention is needed to reduce the prevalence of stunting [12].

The WHO reported that stunting is declining slowly, from 32.4% in 2000 to 21.3% in 2019, and in some regions stunting affects 1 in every 3 children [13, 14]. Slow progress in reducing stunting has been made in Indonesia, from 37.2 to 30.8% in the last 5 years [15]. The stunting rate is relatively high based on the WHO categories of public health significance for stunting (30–39%) [16]. The incidence of stunting in Indonesia is influenced by characteristics of the child (sex; breastfeeding status; early initiation of breastfeeding; infectious diseases, particularly diarrhoea and acute respiratory infections; and birth weight); the household (family size and structure, including mother’s education and knowledge about nutrition and reproductive health, and household and housing characteristics), and the environment (healthcare services and community) [4, 17–19]. Previous studies show that maternal characteristics (health, nutrition, and socio-demography) significantly influence the occurrence of stunting among children less than 5 years old. In general, these studies recommended early integrated interventions to reduce stunting in Indonesia, such as effective education to improve the knowledge, attitudes, and practices of pregnant women regarding nutrition and reproductive health using multi-sectorial approaches. Other scientific evidence shows that more than one-third of mothers do not yet know about stunting, so health promotion and education to improve mothers’ knowledge, attitudes, and practices is needed [20].

Strategies to improve mothers’ knowledge, attitudes, and practices regarding nutrition and reproductive health have consistently contributed to reducing child stunting in Indonesia [20, 21]. Most of the mothers in this country are the primary caregivers for their babies and decide on feeding patterns, immunisation, and health services [4, 17]. Therefore, educational methods as an intervention to improve mothers’ knowledge, attitudes, and practices have been implemented based on the theory of changes behaviour [22, 23]. Research on knowledge, attitude, and behaviour has assessed the success of an educational method by applying a pre-test and post-test research design, such as a quasi-experimental or randomized trial [23]. The interactions between knowledge, attitude, and behaviour initiate a potentially reciprocal and dynamic relationship: knowledge regarding nutrition and reproductive health can inform attitude about that topic, which can influence behaviour [23]. Improving knowledge, attitude, and behaviour during pregnancy is important as it determines post-partum quality of life for the mother and her babies. For example, success of early breastfeeding initiation and exclusive breastfeeding can be determined by mothers’ intention during pregnancy to breastfeed [24–26].

The purpose of this study was to assess the effectiveness of nutrition and reproductive health education of pregnant women in improving knowledge, attitudes, and practices regarding nutrition and reproductive health in Bogor District, in West Java Province, where the stunting rate (about 31%) is higher than the national prevalence [27]. Preliminary studies in this region showed that 29.7% of children were stunted (19% stunted and 10.7% severely stunted). This study used interactive education methods that cover three topics: parenting, balanced diet and immunisation, and reproductive health. Each topic was addressed using different techniques: role-playing, exercises, and fun games supported by interesting props. This educational method could be adopted or modified by the government and other health care providers in an effort to reduce the prevalence of stunting.

## Methods

### Study area and period

The study was conducted in Bogor Regency from August to November 2019. The district is located close to the National Capital as the centre of government, services, and trade, with fairly high development activities. The estimated population of this district is 5,715,009, the highest in West Java Province. The population is 49% female, with an estimated number of 40,896 pregnant women.

Bogor District consists of 40 sub-districts with varying regional morphology, namely lowlands and highlands. Most of the population is poorly educated, and in general, the residents of this district work as entrepreneurs, private or government employees, and labourers, while the majority of mothers are housewives. Moreover, most mothers are the primary caregivers for children [28].

### Design and samples

A quasi-experimental study with a pre-test/post-test design was conducted on pregnant women. The source population in this study was pregnant women in Bogor District, in which the focus locations for stunting consist of 10 villages. Pregnant women selected from four villages were the study population. All eligible women were included in the study, and participants were selected by simple random sampling (SRS) method.

### Inclusion and exclusion criteria

Pregnant women who lived at least 6 months in these villages were included in this study to maintain homogeneity in access to information exposure and health services regarding nutrition and reproductive health. Another inclusion criterion was a maximum gestational age of 27 weeks (end of the second trimester), because the intervention is intended to be implemented before the delivery period. The exclusion criteria were confirmation or diagnosis of serious health problems requiring a special diet and nutritional needs, as well as premature delivery during the data collection period.

### Sample size determination

The sample size was calculated using a formula for the two-sample test of proportions with a one-sided alternative hypothesis, using the following assumptions: 95% confidence level, 82.6% of pregnant women with improved knowledge regarding appropriate dietary practice after receiving nutrition education in the intervention group (*P*_*1*_), and 47.8% of pregnant women with improved knowledge regarding appropriate dietary practice without nutrition education in the control group (*P*_*2*_*)* based on previous study [29], 90% power, 10% contingency for loss to follow-up, and design effect 2. Therefore, using the following formula, the calculated sample size was 194 (97 for each group) pregnant women [30]:

1 $$ n=\frac{{\left\{{z}_{1-\alpha}\sqrt{2\overline{P}\Big(1-\overline{P\Big)}}+{Z}_{1-\beta}\sqrt{P_1\left(1-{P}_1\right)+{P}_2\left(1-{P}_2\right)}\right\}}^2}{{\left({P}_1-{P}_2\right)}^2} $$

### Sampling technique and procedure

**Fig. 1:**
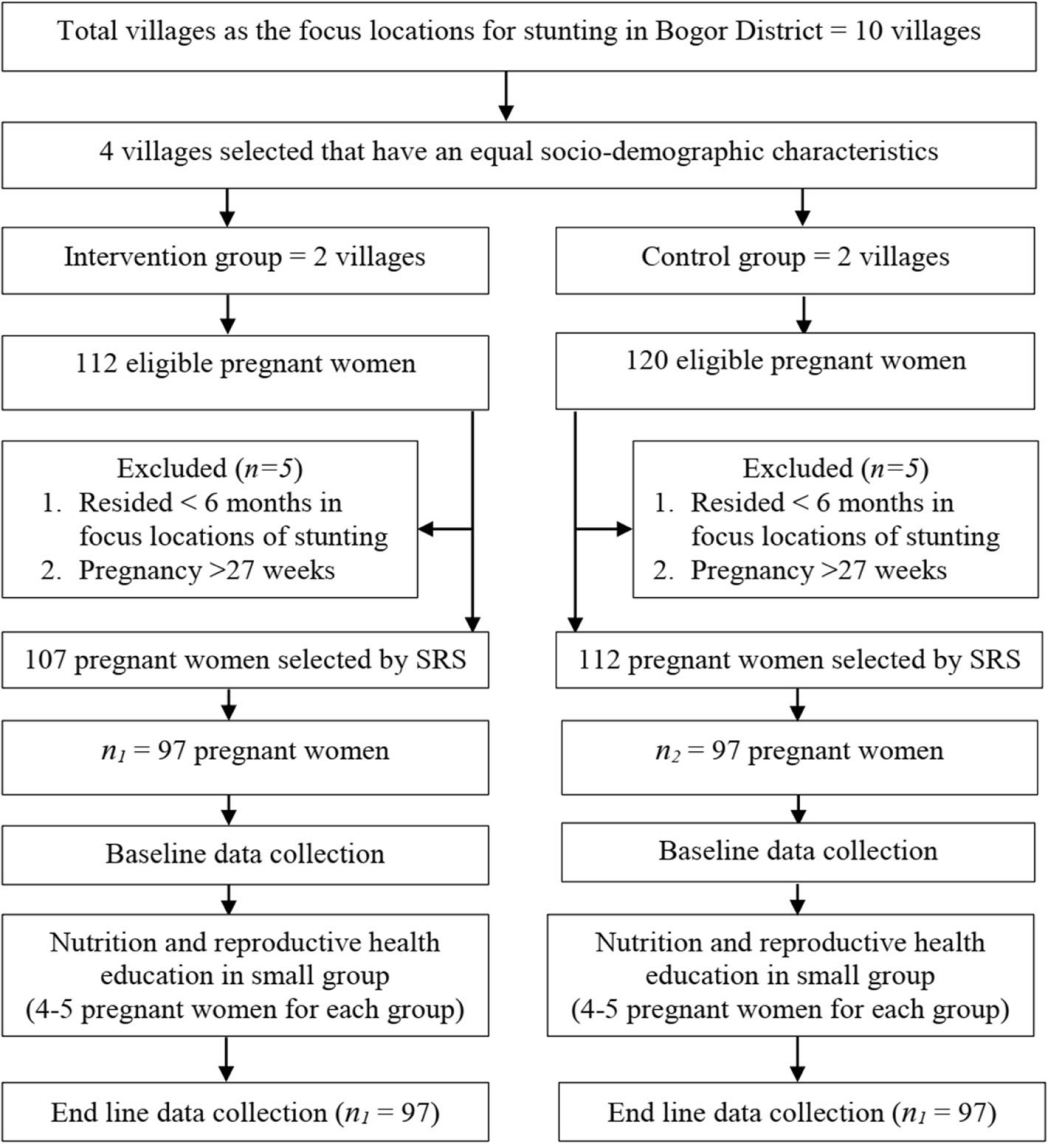
Sampling procedure of pregnant women. SRS = Simple Random Sapling; *n*_*1*_ = sample size for intervention group; *n*_*2*_ = sample size for control group

The sampling procedure is illustrated in Fig. 1. Pregnant women living in four villages, selected from the 10 villages in Bogor District identified as focus locations for stunting in The Bogor District Health Office data, and also recommended as priority areas for intervention, were identified and screened against the inclusion and exclusion criteria. Eligible participants were selected by the simple random sampling method.

### Data collection and measurements

**Fig. 2:**
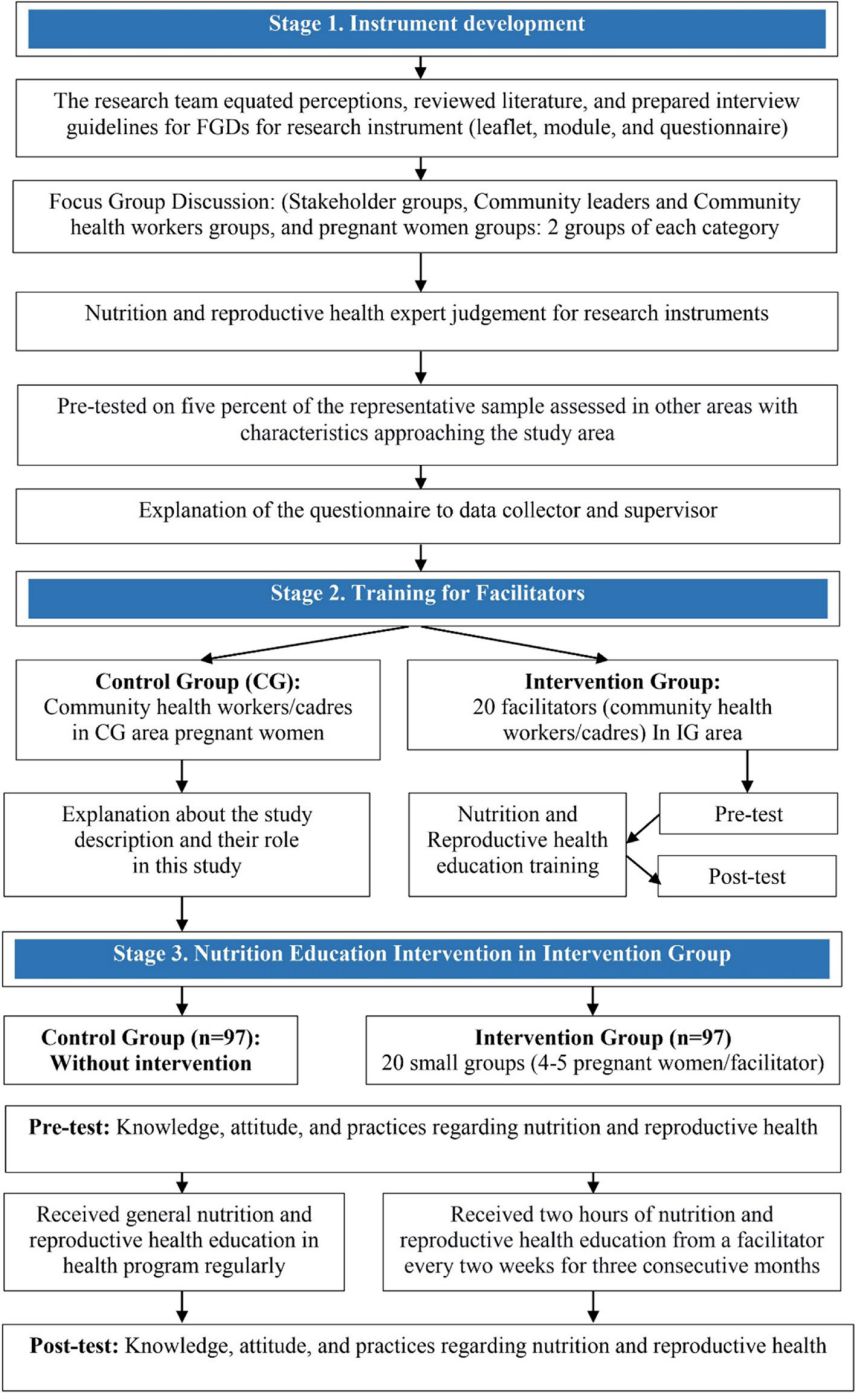
The stages of data collection and measurement. FGD = focus group discussion

This study consists of three stages: 1) instrument development, 2) training for facilitators, and 3) nutrition and reproductive health education of pregnant women in the intervention group (Fig. 2).

#### Instrument development

A focus group discussion (FGD) was conducted to develop a research instrument including leaflet, module, and questionnaire involving stakeholders, community leaders, cadres, and pregnant women. After that, nutrition and reproductive health experts reviewed the research instruments to fulfil the evidence based on the test content. This study also used ‘nutrition discs’, which are educational tools developed and produced by Pergizi Pangan Indonesia (Indonesian Nutrition and Food Expert Association), an organization of nutrition and food experts. Facilitators used the leaflet, module, and nutrition discs to educate pregnant women in the intervention group. The structured questionnaire was applied to collect data on the intervention group and the control group. Maternal socio-demographic characteristics; other information such as obstetric history, nutrition, and reproductive health; and utilisation of health services were collected only at baseline. Knowledge, attitudes, and practices regarding nutritional and reproductive health were collected at both baseline and end line by using 23, 16, and 12 questions, respectively. For knowledge, participants chose one of four answer choices (A, B, C, or D), then were given a score = 1 if the answer is correct, and a score = 0 if the answer is wrong. Items of attitudes were measured using a four-point Likert scale (strongly disagree = 1 through strongly agree = 4). Then, the value of each Likert scale assessed by the participants for each question was summed and the average was calculated. Similarly, items of practices regarding nutrition and reproductive health were collected using a four-point Likert scale (never = 1 through most of the time = 4). Then, each item was summed to obtain an overall score and the average score was calculated.

#### Training for facilitators

The facilitators were cadres or community health workers in the local communities who provided health education to the pregnant woman in the intervention group. They were given training about the health educational skills, methods, and knowledge regarding nutritional and reproductive health. A pre-test/post-test assessment was used to ensure the homogeneity of the facilitators’ skills and knowledge. All facilitators had appropriate socio-demographic characteristics. They are housewives with low socio-economic levels and served as a cadre for at least 1 year.

#### Nutrition and reproductive health education of pregnant women in the intervention group

This education intervention was designed according to the characteristics and information required by pregnant women based on the result of the FGD that involved stakeholders, community leaders, community health workers, and pregnant women (Fig. 2), who provided suggestions so that the educational methods used would be easy to understand and implemented during the pregnancy period to prevent stunting. The majority of pregnant women had low education and socio-economic level in the study area. Their literacy level might have been a barrier to access information regarding nutrition and reproductive health. They are generally housewives who have sufficient time to receive nutrition and reproductive health education for 2 h. These data are reinforced by data obtained from health profile reports and socio-demographics [28]. Advice from nutrition and reproductive health experts also confirms this method. Interactive education can increase participants’ interest and concentration in receiving education. So this method can improve the knowledge, attitudes, and practices regarding nutrition and reproductive health, to prevent stunting since the pregnancy period.

Nutrition and reproductive health education was only given to the intervention group. They were placed in small groups (four or five mothers per group) and received 2 h of nutrition and reproductive health education from a facilitator every 2 weeks for 3 consecutive months. The educational contents and special applied strategies can be seen in Table 1. The education consisted of three sessions that included theoretical (lectures) and practical sessions. The first covered parenting (psycho-emotional and nutritional parenting) and was complemented by role-playing. The second covered nutrition during pregnancy, stunting, and immunity. This session was reinforced by simulation to assess nutritional status and nutritional requirements for the first 1000 days of life. In this session, the facilitator used two packets of nutrition discs, one consisting of eight discs that determine the nutritional status of children based on age groups, and another consisting of eight discs focussed on the needs of balanced nutrition from the gestational period through adolescence (19 years). The third session covered reproductive health education, equipped with the games of myths and facts. The control group received from cadres the usual nutrition and reproductive health education that is provided in the health program regularly every month. In this routine health program, pregnant women are informed about maternal and child health including nutrition and reproductive health, children’s weight and height are measured, and children are given primary immunizations.

**Table 1:** Educational contents and special strategies applied in the education of pregnant women in the intervention group

No.	Educational contents	Special applied strategies^**a**^		Duration
		Theoretical	Practical	
1.	Parenting • Psycho-emotional parenting • Nutritional parenting	Lectures (module, leaflet, flipchart)	Role play (scenario, props, name tags, accessories for each participant)	• 35 min for lectures • 25 min for role play
2.	Nutrition during pregnancy, stunting, and immunity	Lectures (module, leaflet, flipchart)	Simulation (nutrition discs)	• 15 min for lectures • 15 min for simulation
3.	Reproductive health education	Lectures (module, leaflet, flipchart)	Games: myths and facts (list of questions, props)	• 15 min for lectures • 15 min for games
				Total = 120 min

^a^The education is given every 2 weeks for 3 consecutive months. Each meeting involves different material according to the topic in the module

### Quality Assurance of Data Collection

Four women nutritionists acting as data collectors and two public health practitioners as supervisors were given 2 days of training. The questionnaire was pre-tested on 5% of the representative sample assessed in other areas with characteristics similar to those of the study area. Data collectors administered the questionnaire through face-to-face interviews at the pregnant women’s homes and were overseen by supervisors periodically. All questionnaires were verified for completeness and accuracy by data collectors.

### Data processing and analysis

All data in the questionnaire were checked for missing values, including maternal characteristics and knowledge, attitudes, and practices. Furthermore, data were coded and input using SPSS version 20.0. Variables with continuous data, including knowledge, attitudes, and practices scores, were analysed for normality using the Kolmogorov–Smirnov test. Based on the Kolmogorov test, all variables including knowledge, attitudes, and practices showed normal distribution. Descriptive statistics consisting of the mean, standard deviation, and percentage were analysed by univariate analysis, while variables with categorical data were analysed using the chi-square test. A 95% confidence level and a value of *P* < 0.05 were used to assess the statistical significance. Independent t-test was used to see significant differences in pre-test and post-test scores between the intervention group and control group, and paired t-test was used for continuous variables within groups at pre-test and post-test.

### Ethical consideration

Ethics Commission of Health Research of the Faculty of Medicine and Health in Universitas Muhammadiyah Jakarta acceded this study with approval number 001/PE/KE/FKK-UMJ/2019. The Government in Bogor District and each village as the study area also granted permission. The Health Office of Bogor District, the National Population and Family Planning Agency, and community health centres where the villages are located also approved this study. The comfort of pregnant women while participating in the data collection process was prioritised, and the confidentiality of their identity was well guarded.

## Results

### Maternal characteristics

Maternal characteristics included socio-demographic characteristics, obstetric history, information on nutrition and reproductive health, and utilisation of health services as represented in Table 1. A total of 194 pregnant women participated (97 in each group).

#### Socio-demographic characteristics

Most of the participants were of reproductive age (19–35 years). Of 97 participants in each group, about 86.6 and 83.5% of participants were within the age range of 19–35 years in the intervention group (IG) and control group (CG), respectively. Approximately one-third of participants in each group had short stature (height < 150 cm), 33.0% in IG and 28.9% in CG. Almost all participants were housewives in both IG (95.9%) and CG (94.8%). Nearly half of participants graduated from elementary school, 45.4% in IG and 42.3% in CG. Similarly, most of the fathers had an elementary education level in both the IG (79.4%) and CG (70.1%). Family income in the two groups was mostly in the range ≥ of 1,500,000–3,000,000, 59.8 and 62.9% for the GI and CG group. The father and mother made decisions related to health problems together, in both the IG (85.6%) and CG (84.5%). Most of the participants were the original population, both in the IG (78.4%) and CG (79.4%).

#### Obstetric history, information on nutrition and reproductive health, and utilisation of health services

Of 194 participants, most had health insurance provided by the government, about 68.0 and 71.1% in IG and CG, respectively (Table 2). The majority of participants were multigravida (had been pregnant two to four times), 68.0% in IG and 69.1% in CG. Two-thirds had received information about nutrition and reproductive health, in both the IG (69.1%) and CG (67.0%). They obtained general information on antenatal care, at least once in the trimester of pregnancy. Commonly, before the current pregnancy, they used hormonal contraceptive methods such as injection and pills, about 44.4 and 26.8% for IG and 45.4 and 24.7% for CG, respectively. Nevertheless, more than one-third of participants had given birth to their last child at home in both groups. About 35.1 and 30.9% were delivered by traditional birth attendance for the IG and CG, respectively.

**Table 2:** Maternal characteristics in the intervention and control groups in two community health centres in Bogor District (*n*_1_ = *n*_2_ = 97)

Variable	Frequency (n) and percentage (%)				***P*** value
	Intervention		Control		
	group		group		
	n	%	n	%	
**A. Socio-demographic Characteristics**					
**Age**					
19–25 years	41	42.3	36	37.1	0.53
26–35 years	43	44.3	45	46.4	
> 35 years	13	13.4	16	16.5	
**Mother’s height**					
< 150 cm	32	33.0	28	28.9	0.058
150–160 cm	63	64.9	59	60.8	
> 160 cm	2	2.1	10	10.3	
**Mother’s education level**					
Elementary school (≤6 years)	44	45.4	41	42.3	0.51
Junior high school (7–9 years)	38	39.1	39	40.2	
Senior high school (9–12 years)	15	15.5	14	14.4	
College (> 12 years)	0	0.0	3	3.1	
**Mother’s occupation**					
Housewife	93	95.9	92	94.8	0.39
Working Mothers	4	4.1	3	3.1	
**Father’s education**					
Elementary school (≤6 years)	77	79.4	68	70.1	0.48
Junior high school (7–9 years)	18	18.6	25	25.8	
Senior high school (9–12 years)	1	1.0	1	1.0	
College (> 12 years)	1	1.0	3	3.1	
**Father’s occupation**					
Entrepreneur	26	26.8	21	21.7	0.64
Private employees	10	10.3	14	14.4	
Government employees	1	1.0	0	0.0	
Labour	49	50.5	53	54.6	
Others	11	11.3	9	9.3	
**Family income**					
< 1,500,000 IDR	19	19.6	20	20.6	0.19
1,500,000–3,000,000 IDR	58	59.8	61	62.9	
> 3,000,000 IDR	20	20.6	16	16.5	
**Decision maker in the household**					
Father	8	8.2	10	10.3	0.32
Mother	6	6.2	5	5.2	
Father and mother	83	85.6	82	84.5	
**Residence status**					
Original population	76	78.4	77	79.4	0.72
Migrants	21	21.6	20	20.6	
**B. Obstetric History, Information of Nutrition and Reproductive Health, and Utilisation of Health Services**					
**Health insurance**					
No	31	32.0	28	28.9	0.34
Yes	66	68.0	69	71.1	
**Parity**					
Primigravida (pregnant for the first time)	27	27.9	20	20.6	0.26
Multigravida (has been pregnant 2–4 times)	66	68.0	67	69.1	
Grande multigravida (has been pregnant ≥5 times)	4	4.1	10	10.3	
**Exposure to nutrition and reproductive health information**					
No	30	30.9	32	33.0	0.43
Yes	67	69.1	65	67.0	
**Contraceptive methods (before the current pregnancy)**					
Do not use any method of contraception	21	21.7	20	20.6	0.39
Natural birth control	4	4.1	5	5.2	
Injection	43	44.3	44	45.4	
Pills	26	26.8	24	24.7	
Other contraception (IUD/vaginal ring/condoms)	3	3.1	4	4.1	
**Place of delivery (the last childbirth)**					
Have never been pregnant before	27	27.8	20	20.6	0.09
At home	35	36.1	35	36.1	
Health facilities (birth centre/hospital)	35	36.1	42	43.3	
**Birth attendants (the last childbirth)**					
Have never been pregnant before	27	27.8	20	20.6	0.31
Traditional birth attendant (TBA)	34	35.1	30	30.9	
Skilled birth attendant (doctor, nurse, midwife)	36	37.1	47	48.5	

### Effect of nutrition and reproductive health intervention

Table 3 presents that the overall mean nutritional and reproductive health knowledge scores were significantly improved (*P* < 0.001) from 55.1 to 83.1 in IG. The paired t-test indicated that there was a significant difference (*P* < 0.05) between pre-test and post-test in IG. Similarly, the overall mean attitudes score was significantly different between pre-test and post-test (*P* < 0.05), from 40.2 to 49.0, in IG. The highest attitudes score was an increase in nutritional parenting (3.4) from 10.2 to 13.8 and the lowest in reproductive health (2.4) from 7.8 to 10.2. The paired t-test also showed an increased overall mean practices score in IG (*P* < 0.05), from 36.2 to 40.2. The independent t-test indicated that there was a significant difference in all aspects of knowledge, attitudes, and practices in the post-test between IG and CG (*P* < 0.05), but there was no significant difference at pre-test (*P* > 0.05).

**Table 3:** Effects of nutrition and reproductive health interventions on the knowledge, attitudes, and practices of pregnant women (*n*_1_ = *n*_2_ = 97)

Variable	Group	Research period (Mean score ± SD)		***P*** value^**a**^
		Pre-test	Post-test	
**Knowledge**				
*Parenting*				
• Psycho-emotional parenting (5 items)	Intervention	53.4 ± 3.8	83.1 ± 5.3	< 0.001
	Control	53.2 ± 6.2	53.8 ± 5.7	0.78
	*P* value^b^	0.96	< 0.001	
• Nutritional parenting (5 items)	Intervention	62.1 ± 5.4	86.6 ± 4.8	< 0.001
	Control	62.4 ± 4.3	62.9 ± 3.9	0.51
	*P* value^b^	0.42	< 0.001	
*Nutrition during pregnancy, stunting, and immunity (8 items)*	Intervention	54.9 ± 4.4	85.0 ± 2.6	< 0.001
	Control	55.3 ± 3.5	56.0 ± 3.8	0.28
	*P* value^b^	0.65	< 0.001	
*Reproductive health (5 items)*	Intervention	49.9 ± 3.7	77.5 ± 5.6	< 0.001
	Control	50.1 ± 4.2	50.9 ± 4.8	0.31
	*P* value^b^	0.89	< 0.001	
Overall knowledge score (23 items, multiple choice questions)	Intervention	55.1 ± 4.6	83.1 ± 5.2	< 0.001
	Control	55.3 ± 4.2	55.9 ± 3.4	0.26
	*P* value^b^	0.72	< 0.001	
**Attitude**				
*Parenting*				
• Psycho-emotional parenting (4 items)	Intervention	9.6 ± 1.7	12.8 ± 2.9	< 0.001
	Control	9.3 ± 4.4	9.5 ± 3.4	0.22
	*P* value^b^	0.49	< 0.001	
• Nutritional parenting (4 items)	Intervention	10.2 ± 6.1	13.6 ± 4.4	< 0.001
	Control	10.6 ± 4.8	10.8 ± 3.2	0.67
	*P* value^b^	0.35	< 0.001	
*Nutrition during pregnancy, stunting, and immunity* (4 items)	Intervention	9.6 ± 3.6	12.2 ± 2.8	< 0.001
	Control	9.7 ± 3.4	9.8 ± 3.6	0.49
	*P* value^b^	0.54	< 0.001	
*Reproductive health* (4 items)	Intervention	7.8 ± 4.9	10.2 ± 2.7	0.003
	Control	7.5 ± 5.5	7.5 ± 4.8	0.98
	*P* value^b^	0.46	< 0.001	
Overall attitude score (16 items, 4 point Likert scale)	Intervention	40.2 ± 4.2	49.0 ± 4.5	< 0.001
	Control	40.1 ± 3.8	40.5 ± 5.2	0.25
	*P* value^b^	0.82	< 0.001	
**Practices**				
Psycho-emotional parenting during pregnancy (4 items)	Intervention	9.0 ± 3.6	12.2 ± 6.7	< 0.001
	Control	8.8 ± 4.7	9.0 ± 4.6	0.62
	*P* value^b^	0.66	< 0.001	
Dietary practices (4 items)	Intervention	9.2 ± 5.2	12.9 ± 4.2	< 0.001
	Control	9.1 ± 3.9	9.4 ± 3.6	0.29
	*P* value^b^	0.78	< 0.001	
Reproductive health (4 items)	Intervention	7.4 ± 4.1	10.4 ± 5.4	0.010
	Control	7.2 ± 6.4	7.4 ± 3.1	0.46
	*P* value^b^	0.58	0.002	
Overall practices score (12 items, 4 point Likert scale)	Intervention	36.2 ± 4.3	40.2 ± 4.9	< 0.001
	Control	36.0 ± 6.2	36.3 ± 6.8	0.33
	*P* value^b^	0.45	< 0.001	

^a^paired t-test

^b^independent t-test

### Nutrition and reproductive health knowledge of pregnant women

The knowledge of parenting consists of psycho-emotional parenting and nutritional parenting. In psycho-emotional parenting knowledge, almost all of the participants (93.8%) in the IG knew the consequences of not providing psycho-emotional parenting during pregnancy for the growth and development of the baby at the post-test (Table 4). In the nutritional parenting knowledge, almost 100% of participants knew the dietary sources of macronutrient- and micronutrient-rich foods for babies more than 6 months old (95.9 and 96.9%, respectively). There was a significant difference between pre-test and post-test in IG (*P* < 0.001) for each question in this aspect. The participants’ knowledge of nutrition during pregnancy, stunting, and immunity improved after education. Almost 100% of participants (92.8%) knew the dietary sources of macronutrient-rich foods (92.8%), the signs and symptoms of stunting (94.8%), and the benefits of immunisation in childcare (92.8%). Similarly, their reproductive health knowledge also improved at the post-test. As much as 92.8% of participants knew the benefits of antenatal care for maternal and babies’ health. The paired t-test indicated that nutrition and reproductive health knowledge of participants were significantly (*P* < 0.001) increased after education in IG, but there was no significant difference in CG (*P* > 0.05) in all questions.

**Table 4:** Nutrition and reproductive health knowledge of pregnant women in the intervention and control groups (*n*_1_ = *n*_2_ = 97)

Knowledge variable	Research period	Correct answer (Frequency and percentage)		***P*** value
		Intervention group	Control group	
***Parenting***				
*•* **Psycho-emotional parenting**				
The benefits of psycho-emotional parenting during pregnancy for foetus and mother	Pre-test	45 (46.4)	44 (45.4)	0.68
	Post-test	75 (77.3)	44 (45.4)	< 0.001
Appropriate practice of psycho-emotional parenting during feeding period	Pre-test	34 (35.1)	36 (37.1)	0.24
	Post-test	73 (75.3)	37 (38.1)	< 0.001
Ways to improve ‘bonding’ between mother, foetus, and father during pregnancy	Pre-test	60 (61.9)	59 (60.8)	0.74
	Post-test	88 (90.7)	60 (61.9**)**	< 0.001
The benefits of exclusive breastfeeding towards the baby’s psycho-emotional development	Pre-test	50 (51.5)	50 (51.5)	0.98
	Post-test	76 (78.4)	50 (51.5)	< 0.001
The consequences of not providing psycho-emotional parenting during pregnancy for the baby’s growth and development	Pre-test	70 (72.2)	69 (71.1)	0.84
	Post-test	91 (93.8)	70 (72.2)	< 0.001
*•* **Nutritional parenting**				
The best nutrition source for babies 0–6 months old	Pre-test	40 (41.2)	42 (43.3)	0.37
	Post-test	72 (74.2)	43 (44.3)	< 0.001
Timing of complementary feeding for babies	Pre-test	76 (78.4)	75 (77.3)	0.69
	Post-test	91 (93.8)	75 (77.3)	< 0.001
Dietary sources of macronutrient-rich foods for babies > 6 months old	Pre-test	77 (79.4)	77 (79.3)	0.007
	Post-test	93 (95.9)	78 (80.4)	0.009
Dietary sources of micronutrient-rich foods for babies > 6 months old	Pre-test	78 (80.4)	78 (80.4)	0.98
	Post-test	94 (96.9)	78 (80.4)	0.001
Appropriate food processing techniques according to baby’s age	Pre-test	30 (30.9)	31 (31.9)	0.85
	Post-test	70 (72.2)	31 (31.9)	< 0.001
***Nutrition during pregnancy, stunting, and immunity***				
Understanding of balanced diet	Pre-test	55 (56.7)	57 (58.8)	0.26
	Post-test	83 (85.6)	58 (59.8)	< 0.001
The benefit of a balanced diet during pregnancy for foetus and mother	Pre-test	57 (58.8)	56 (57.7)	0.39
	Post-test	82 (84.5)	58 (59.8)	< 0.001
Dietary sources of macronutrient-rich foods	Pre-test	60 (61.9)	59 (60.8)	0.28
	Post-test	90 (92.8)	60 (61.9)	< 0.001
Dietary sources of micronutrient- rich foods	Pre-test	59 (60.8)	60 (61.9)	0.47
	Post-test	82 (84.5)	61 (62.9)	< 0.001
Understanding of stunting	Pre-test	58 (59.8)	57 (58.8)	0.75
	Post-test	90 (94.8)	57 (58.8)	< 0.001
Long-term consequences of stunting in early life	Pre-test	45 (46.4)	48 (49.5)	0.14
	Post-test	79 (81.4)	50 (51.5)	< 0.001
Synergetic effect of nutrition and infection towards stunting	Pre-test	41 (42.3)	40 (41.2)	0.42
	Post-test	80 (82.5)	41 (42.3)	< 0.001
Benefits of immunisation in childcare	Pre-test	67 (69.1)	66 (68.0)	0.56
	Post-test	90 (92.8)	66 (68.0)	< 0.001
***Reproductive health***				
Benefits of family planning for maternal and babies’ health	Pre-test	46 (47.4)	46 (47.4)	0.35
	Post-test	78 (80.4)	48 (49.5)	< 0.001
Appropriate contraception method for postpartum mothers	Pre-test	21 (21.7)	22 (22.7)	0.44
	Post-test	75 (78.4)	22 (22.7)	< 0.001
Benefits of antenatal care for maternal and babies’ health	Pre-test	70 (72.2)	71 (73.2)	0.52
	Post-test	90 (92.8)	72 (74.2)	< 0.001
Appropriate breast healthcare practice during pregnancy and lactation	Pre-test	53 (54.6)	51 (52.6)	0.12
	Post-test	77 (79.4)	51 (52.6)	< 0.001
Appropriate women’s reproductive (sexual health/reproductive organ hygiene) health care practice	Pre-test	52 (53.6)	53 (54.6)	0.26
	Post-test	79 (81.4)	54 (55.7)	< 0.001

## Discussion

The failure of foetal growth during pregnancy is significantly related to stunted child growth [31]. Pregnant women have central roles in achieve optimal growth during this critical period [4, 31]. The 194 pregnant women who participated in this study have a high risk of having stunted children, generally. They live in rural areas with low socio-economic levels that are related to a lack of food availability in the household [32, 33]. Another study among pregnant women in rural Punjab showed that education and parity were significantly associated with knowledge, attitude, and dietary practices [34, 35]. The WHO reported that mothers with low income and a low level of education experience more difficulty affording adequate food to provide a nutritious and diverse diet [36]. The findings of this study also indicate that about one-third of pregnant women (33.0 and 28.9% in IG and CG, respectively) also have a short stature (> 150 cm), which may increase risk of having stunted children [37–39]. A 19-year-old woman with a height at least two standard deviations below the average (> 150 cm) has short stature, according to the WHO provision [40]. Javid and Pu showed in the Pakistan Demographic and Health Survey of 2012–13 that short-stature mothers (height > 150 cm) were about 2.0 times more likely to have a stunted child compared to tall-stature mothers [38]. Participants in this study also have not utilised health services optimally. More than one-third of those who have given birth delivered their babies at home and were helped by traditional birth attendants. They did not receive adequate health care in the early life of the baby, which is a crucial stage associated with pregnancy outcomes. This condition indicates a low quality of maternal health care, which may significantly affect child stunting [41, 42].

Knowledge, attitude, and practices regarding nutrition and reproductive health are the main factors that can influence pregnancy outcomes [43, 44]. The finding of this study indicates that participants have a lack of knowledge, attitudes, and practices regarding nutrition and reproductive at baseline. In this study, the knowledge, attitudes, and practices mean scores were significantly (*P* < 0.001) improved after education in the IG*.* In the CG, there was no significant difference (*P* > 0.05) in mean knowledge, attitudes, and practices scores regarding nutrition and reproductive health between pre-test and post-test. This study also shows that education intervention effectively provides a significant (*P* < 0.05) difference in the mean knowledge, attitudes, and practices scores between the IG and CG at the end line: about 82.1 and 55.9, 49.0 and 40.5, and 40.2 and 36.3, respectively of the IG and CG between pre-test and post-test. Similarly, a cluster randomized control trial study among pregnant women in Northeast Ethiopia showed that nutrition education significantly improved (*P* < 0.001) mean nutritional knowledge scores in the intervention group, from 6.9 at baseline to 13.4 after nutrition education. There was a significant difference (*P* < 0.001) in mean nutritional knowledge scores between the intervention group and control group at baseline. The study also shows that proportion of healthy dietary practices was significantly different (*P* < 0.001) between pregnant women who were given nutrition education in the intervention group compared to the control group at the end line [29]. A study among pregnant women in Addis Ababa showed that nutrition knowledge scores improved after the nutrition education program from 53.9 to 97.0%, whereas dietary practice scores during pregnancy increased from 46.8 to 83.7% [45]. These studies reinforce the evidence that nutrition education has a positive effect on improving the knowledge, attitudes, and practices scores among pregnant women.

Nutrition and reproductive health education is a specific intervention in The Global Nutrition target in 2025 [7] to reach a 40% reduction in the number of children under 5 years old who are stunted. This intervention can be delivered effectively through community health workers who have a high potential to improve maternal and child health among the hard-to-reach population, particularly in rural areas [13]. According to the WHO recommendation, that method was applied in this study. The community health workers as facilitators delivered interactive nutrition and reproductive health education. They actively visited the homes of eligible pregnant women, formed groups, and conducted the education in small groups. This method has been proven to improve the knowledge, attitudes, and practices of pregnant women regarding nutrition and reproductive health. The intervention given in this study is different from the methods that have used done before both in educational content and applied strategies, although it has approximately the same effectiveness. The study in West Gojjam Zone, Ethiopia, showed that pregnant women who received nutrition education were 2.02 times more likely to improve their dietary practices compared with those who did not receive nutrition education [46]. The educational method used in the West Gojjam zone study was nutrition counselling provided by health workers, which would be difficult to apply in this study. The number of health workers such as midwives, nutritionists, or health promoters in rural areas is limited. There is more potential to optimise the role of cadres or community health workers as facilitators in providing nutrition and reproductive health education to pregnant women. This allows the intervention to be given continuously during the first 1000 days of life to prevent stunting. A study in Bangladesh showed that maternal counselling using a framework of essential health care (EHC) plus nutrition counselling could improve knowledge and dietary practices of child feeding to reduce stunting prevalence effectively [2]. However, with this method, education is given passively. The educator waits for participants to obtain EHC and nutrition counselling. Less interactive methods can lead to potential saturation for participants in obtaining an education. Furthermore, proper knowledge and dietary practices simultaneously influence gestational weight gain, degrade the risk of anaemia in the last trimester of pregnancy, improve the baby’s birth weight, and reduce the risk of preterm birth [47]. This study strengthens scientific evidence that nutrition and reproductive health education during pregnancy can improve the knowledge, attitudes, and practices of pregnant women, which contribute to increased maternal and neonatal health and reduce childhood stunting [29, 31, 48, 49].

Nutrition and reproductive health knowledge of pregnant women related to stunting improved significantly by education intervention [29, 41]. A formative research study conducted in 10 provinces in Indonesia that implemented the National Nutrition Communication Campaign (NNCC) showed that only 2.1% of 3150 mothers had known about stunting, and about two-thirds of them assumed that stunting was caused by heredity [20]. Nevertheless, a study among women of child-bearing age in Lagos State, Nigeria, reported that 61.89 and 86.89% of them had accomplished knowledge and positive attitude regarding nutrition, respectively [49]. The finding of this study shows that the number of participants who answered correctly for all questions increased significantly (*P* < 0.001) in the IG. In parenting knowledge, almost 100% of participants knew the ways to improve ‘bonding’ between mother, foetus, and father during pregnancy (90.7%) and the consequences of not providing psycho-emotional parenting during pregnancy for the baby’s growth and development (93.8%). A previous study showed that maternal depression was associated with child stunting and psychological and intellectual development. The lack of psycho-emotional parenting during pregnancy weakens ‘mother-child’ attachment that affects the nutritional status and development of children [50, 51]. In nutrition parenting, almost 100% of participants correctly answered the question about the timing of complementary feeding for babies (93.8%), dietary sources of macronutrient-rich foods for babies > 6 months old (95.9%), and dietary sources of micronutrient-rich foods for babies > 6 months old (96.9%). Mistry et al. showed that maternal counselling was associated with improving feeding practices in the early life of a child, which decreased stunting prevalence significantly [2]. The participants improved their knowledge about nutrition during pregnancy, stunting, and immunity after education. Most of the participants knew about a balanced diet (85.6%), the benefit of a balanced diet during pregnancy for foetus and mother (84.55%), dietary sources of macronutrient-rich foods (92.8%),stunting (94.8%), the synergetic effect of nutrition and infection towards stunting (82.5%), and the benefits of immunisation in childcare (92.8%). A study in Dissie Town, Northeast, Ethiopia, showed that the number of participants who answered correctly increased after nutrition education. Almost all of the participants knew about a balanced diet (95.7%), the benefit of a balanced diet for foetus and mother (89.9%), and the synergetic effect between nutrition and infection (97.1%). Also, all participants (100%) knew about the dietary sources of macronutrient- and micronutrient-rich foods [29]. Generally, the increase in the number of participants who answered correctly in Dissie Town is higher than in this study. The participants live in urban areas, so they get better access to health facilities. They also have a higher socio-economic status, including education, occupation, and family income. This study also shows an increase in the number of participants who answered correctly in reproductive health knowledge: 92.8% of participants knew the benefits of antenatal care for maternal and baby health after education. Similarly, a quasi-experimental study in Brebes District, Central Java in Indonesia, showed that reproductive health education improved knowledge among brides and grooms [52]. Additional evidence in Somalia shows that they have a lack of knowledge about reproductive health that confirmed the need for proper nutrition education [53]. Reproductive health during pregnancy is fundamental to ensuring all women have access to respectful and high-quality maternity care to increase maternal health and pregnancy outcomes [54].

This study contributes to increasing intensive nutrition and reproductive health education efforts implemented in the wider community. Such studies provide scientific evidence as consideration for policymakers, researchers, program practitioners and implementers, non-governmental organisations, health workers, community health workers, and the entire community to improve knowledge, attitudes, and practices regarding nutrition and reproductive health, in order to reduce the prevalence of stunting from 27.7 to 14% in 2024 as the national target in Indonesia and achieve The Global Nutrition target in 2025 of 40% reduction in the number of children under 5 years old who are stunted.

## Conclusion

Nutrition and reproductive health education delivered by community health workers as agents of behaviour change was effective in improving knowledge, attitude, and practices regarding nutrition and reproductive health in pregnant women in the study area. The pregnant women had good knowledge, attitudes, and practices regarding psycho-emotional parenting, nutrition parenting, nutrition during pregnancy, stunting, immunity, and reproductive health. Similar strategic efforts could help to reduce the prevalence of stunting in the first 1000 days of life.

## Recommendations

Cross-sectoral cooperation, especially collaboration between the health office and the National Population and Family Planning Agency, and optimisation of community empowerment are needed to strengthen education on nutrition and reproductive health of pregnant women sustainably. The education methods also need to be adapted to the local culture to help community health workers carry out health promotion and ensure it is readily accepted by pregnant women in order to accelerate the improvement of knowledge, attitudes, and practices regarding nutrition and reproductive health. Also, the process of continuous supervision by health workers, such as midwives, nutritionists, public health workers, or other health practitioners, to ensure that nutrition and reproductive health education programs continue through the first 2 years of a baby’s life, as a critical period of infant growth and development, could help to prevent stunting.

## Data Availability

The datasets used and/or analysed during the current study are available from the corresponding author on reasonable request.
